# The AMPK system of salmonid fishes was expanded through genome duplication and is regulated by growth and immune status in muscle

**DOI:** 10.1038/s41598-019-46129-4

**Published:** 2019-07-08

**Authors:** Dwight R. Causey, Jin-Hyoung Kim, Robert H. Devlin, Samuel A. M. Martin, Daniel J. Macqueen

**Affiliations:** 1 School of Biological Sciences, University of Aberdeen, Aberdeen, UK; 2 Fisheries and Oceans Canada, West Vancouver, British Columbia, V7V 1N6 Canada; 3 Present Address: Korea Polar Research Institute (KOPRI), Yeonsu-gu, Incheon 21990 Korea; 4 Present Address: The Roslin Institute and Royal (Dick) School of Veterinary Studies, University of Edinburgh, Edinburgh, UK

**Keywords:** Animal physiology, Evolutionary biology, Homeostasis

## Abstract

5′adenosine monophosphate-activated protein kinase (AMPK) is a master regulator of energy homeostasis in eukaryotes. This study identified expansions in the AMPK-α, -β and -γ families of salmonid fishes due to a history of genome duplication events, including five novel salmonid-specific AMPK subunit gene paralogue pairs. We tested the hypothesis that the expanded AMPK gene system of salmonids is transcriptionally regulated by growth and immunological status. As a model, we studied immune-stimulated coho salmon (*Oncorhynchus kisutch*) from three experiment groups sharing the same genetic background, but showing highly-divergent growth rates and nutritional status. Specifically, we compared wild-type and GH-transgenic fish, the latter achieving either enhanced or wild-type growth rate via ration manipulation. Transcript levels for the fifteen unique salmonid AMPK subunit genes were quantified in skeletal muscle after stimulation with bacterial or viral mimics to alter immune status. These analyses revealed a constitutive up-regulation of several AMPK-α and -γ subunit-encoding genes in GH-transgenic fish achieving accelerated growth. Further, immune stimulation caused a decrease in the expression of several AMPK subunit-encoding genes in GH-transgenic fish specifically. The dynamic expression responses observed suggest a role for the AMPK system in balancing energetic investment into muscle growth according to immunological status in salmonid fishes.

## Introduction

5′adenosine monophosphate-activated protein kinase (AMPK) is the primary sensor of cellular energetic status in eukaryotes, including vertebrates^1,2^. During cellular energetic stress, when AMP and ADP levels are high and ATP levels are low, AMPK is activated, leading to stimulation of pathways that produce ATP, while inhibiting anabolic processes that consume ATP. AMPK is activated by an increase in the AMP:ATP ratio, and its numerous actions are regulated through allosteric activation, phosphorylation by upstream kinases, inhibition of dephosphorylation and calcium signalling^3,4^. AMPK is involved in many signalling pathways, with roles including stimulation of catabolic pathways (e.g. glucose uptake, glycolysis, fatty acid uptake and mitochondrial biogenesis) and inhibition of anabolic pathways (e.g. protein synthesis, cholesterol synthesis, triglyceride synthesis and gluconeogenesis). AMPK is composed of three subunits that are highly conserved, namely the catalytic α-subunit and the regulatory β- and γ-subunits^5–7^. Human genomes contain seven different genes encoding unique AMPK subunits (two AMPK-α, two AMPK-β and three AMPK-γ subunits), and orthologues of these three subunits are present across the eukaryotic kingdoms^6,8,9^.

Previous studies have demonstrated the existence of a functional AMPK system in teleost fishes^6,8,10–12^. Consistent with the teleost-specific whole genome duplication (tsWGD) event (e.g.^13,14^), an expansion in AMPK subunit encoding genes has been reported in teleosts^6,8^. This includes duplications of the β1, γ2 and γ3 subunits resulting in a total of ten AMPK subunits in some teleosts (e.g. zebrafish)^8^. The functional roles and regulation of AMPK have been explored in several teleosts under a range of physiological conditions including crucian carp (*Carassius carassius*)^15^, goldfish (*Carassius auratus*)^16^, zebrafish (*Danio rerio*)^17^, fine flounder (*Paralichthys adspersus*)^10^, brown trout (*Salmo trutta*)^11^ and rainbow trout (*Oncorhynchus mykiss*)^6,12^. For example, the study in brown trout highlighted importance for AMPK in skeletal muscle glucose metabolism^11^.

Such past studies of fishes have generally considered changes in AMPK total protein abundance and/or phosphorylation status using antibody-based approaches^6,10,11,15–17^, which may not distinguish closely-related genes. Other studies have targeted specific AMPK genes at the mRNA level^6,15,16^. However, the presence of an additional ancestral salmonid-specific WGD (ssWGD) event that occurred ~95 Mya^18,19^, and from which 50–60% of all genes are retained as functional paralogues^20,21^, suggests the salmonid AMPK system may contain previously uncharacterized genes. Salmonid paralogues are widely differentially expressed under different physiological conditions (e.g.^22–24^), necessitating efforts to distinguish them in molecular investigations. The availability of high-quality genomes for several salmonid species (see^25^) can be used to aid identification of salmonid-specific AMPK subunit paralogues, which represents one of the objectives of this study.

Considering its role in energy homeostasis, AMPK likely represents a key player in the coordination of energetic allocation into different physiological functions. Growth and immunity are two energy demanding systems, each essential to survival and fitness. Consequently, strong interdependencies have evolved to manage the balance of investment into growth and immunity^26^, which in fishes may be underpinned by cross-talk between conserved growth and immune pathways (e.g.^22,23,27^). AMPK is hypothesised here to have roles in cross-talk between the growth and immune systems according to the intrinsic and external physiological conditions.

Growth hormone (GH) transgenic salmon provide an ideal model to explore the role of AMPK in cross-talk between the immune and growth systems. In this study, we exploit an established experimental design^27^, where GH-transgenic and wild-type coho salmon were subjected to immune stimulation, allowing the impact of growth rate and energetic status on immune function to be investigated. Skeletal muscle was selected for analysis because of its importance for energetic investment and storage^28^. Salmonids with a GH transgene inserted into a wild-type genetic background overexpress GH at levels up to 40-fold higher than wild-type^29^. This leads to an large increase in the endocrine production of insulin-like growth factor-I (IGF-I)^30^, a master anabolic hormone secreted from liver in response to GH, and consequent elevation of feed intake (~3-fold) and growth^29,31^. As growth can only occur with sufficient resources, providing GH-transgenic salmon with a dietary ration satiating for wild-type fish restricts growth rate to near wild-type^30,32^. This restricted ration GH-transgenic experimental group helps to separate the effects of GH from its downstream impacts on accelerated growth^27^.

To date, AMPK has been studied in many transgenic model systems (e.g.^33^), but as far as we are aware, existing literature has yet to explore the regulation of AMPK in GH-transgenic animals, nor in the context of interactions between growth and immune status. The first aim of this study was to characterize the complete set of AMPK subunit genes retained in the salmonid lineage from the tsWGD and ssWGD events. The second aim was to test the hypothesis that AMPK is involved in cross-talk between the growth and immune systems, by measuring the transcriptional responses of AMPK subunit genes following systematic stimulation with mimics of bacterial and viral infections. By comparing such responses in wild-type and GH-transgenic salmon achieving different growth rates, we aimed to disentangle the natural response of the AMPK system to immune stimulation from alterations specifically associated with accelerated growth.

## Results

### Phylogenetic analysis of AMPK subunits and nomenclature system

**Figure 1:**
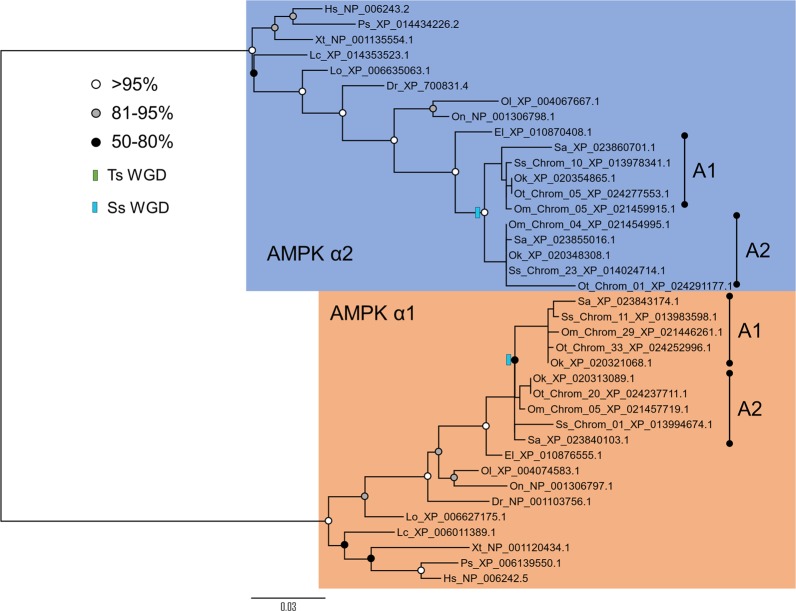
Maximum likelihood phylogenetic analysis of AMPK-α subunit sequences from: human *Homo sapiens* (“Hs”), Chinese softshell turtle *Pelodiscus sinensis* (“Ps”), western clawed frog *Xenopus tropicalis* (“Xt”), West Indian Ocean coelacanth *Latimeria chalumnae* (“Lc”), spotted gar *Lepisosteus oculatus* (“Lo”, sister lineage to teleosts that did not undergo tsWGD)^37^, zebrafish *Danio rerio* (“Dr”), Japanese rice fish *Oryzias latipes* (“Ol”), tilapia *Oreochromis niloticus* (“On”), northern pike *Esox lucius* (“El”, a sister lineage to salmonids that did not undergo ssWGD^25^, Arctic charr *Salvelinus alpinus* (“Sa”), Atlantic salmon *Salmo salar* (“Ss”), rainbow trout *Oncorhynchus mykiss* (“Om”), Chinook salmon *Oncorhynchus tshawytscha* (“Ot”) and coho salmon *Oncorhynchus kisutch* (“Ok”). The tree is annotated to show WGD events in the teleost (“TsWGD”) and salmonid ancestor (“SsWGD”). Bootstrap branch support values are shown as circles on each node. Chromosomal locations for salmonid genes are provided when available. Accessions numbers are provided for all sequences.

**Figure 2:**
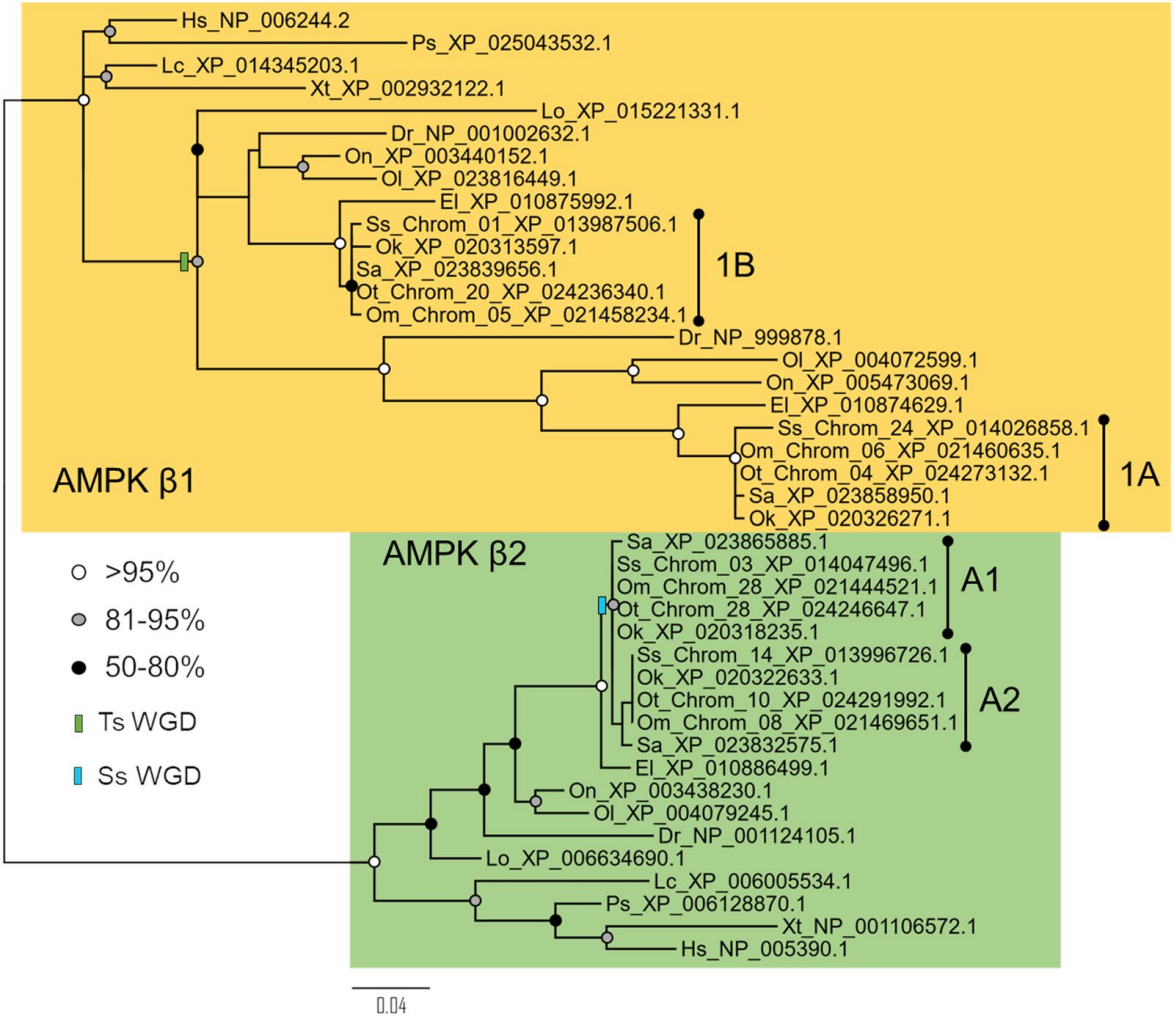
Maximum likelihood phylogenetic analysis of the AMPK-β subunit. Details of species abbreviations and other annotations as in Fig. 1 legend.

**Figure 3:**
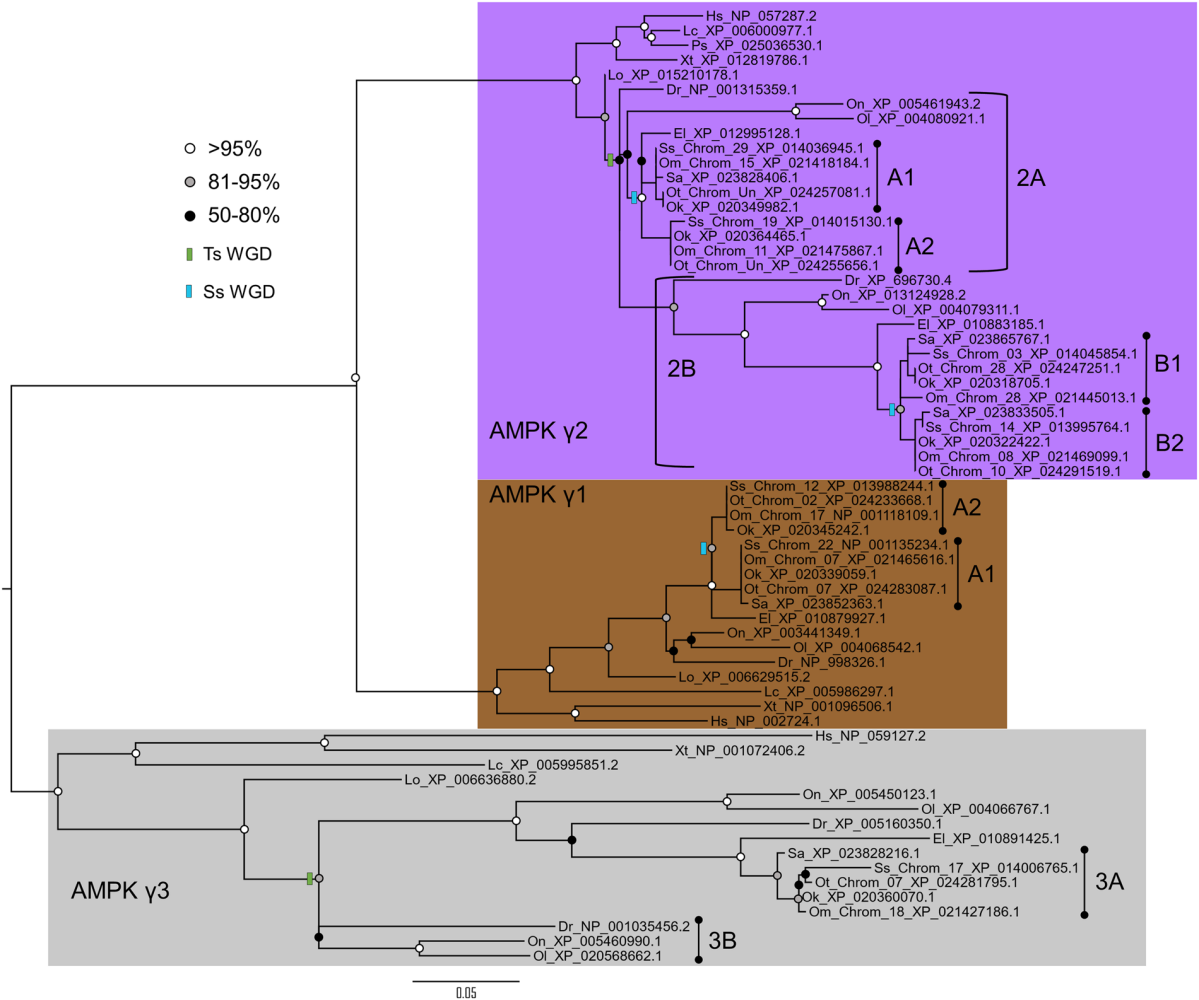
Maximum likelihood phylogenetic analysis of the AMPK-γ subunit. Details of species abbreviations and other annotations as in Fig. 1 legend.

Three separate maximum likelihood (ML) phylogenetic analyses of amino acid sequence were performed to reconstruct the evolutionary history of the AMPK-α, -β and –γ subunit families (Figs 1–3, respectively). By including sequences gathered from multiple teleost lineages, including salmonids, these analyses accommodated the presence of paralogues retained from the tsWGD and ssWGD events. For the included salmonid species, the databases searched contain RefSeq genes predicted within reference genomes assembly for each species, namely: Atlantic salmon (*Salmo salar*)^20^, rainbow trout (*Oncorhynchus mykiss*) (unpublished; NCBI accession: GCA_002163495), Chinook salmon (*O*. *tshawytscha*)^34^, coho salmon (*O. kitsuch*) (unpublished; NCBI accession: GCA_002021735.1) and Arctic charr (*Salvelinus alpinus*)^35^. For all AMPK genes, we employed a standard nomenclature system advocated elsewhere^36^ with putative paralogues retained from the teleost WGD named “gene A” and “B”. In cases where there was no evidence for the retention of teleost paralogues, we used “gene A”. We gave salmonid-specific paralogues a sub-annotation of “1” or “2” (e.g. “gene A1” and “A2”)^36^.

### Novel salmonid paralogues of AMPK-α

The AMPK-α tree split into two clades (AMPK-α1 and -α2), represented by all included vertebrate species, as reported elsewhere^6,8^ (Fig. 1). In both clades, the branching of represented ray-finned fishes follows expected species relationships, assuming a lack of retention of teleost-specific paralogues, with spotted gar (*Lepisosteus oculatus*) being the sister clade to teleosts^37^. However, in both the AMPK-α1 and -α2 clades, the represented salmonid sequences are split across distinct clades represented by different salmonid species (Fig. 1). Within the AMPK-α2 clade, the salmonid sequences split into two monophyletic clades (Fig. 1). However, in the AMPK-α1 clade, the salmonid sequences did not split cleanly into two monophyletic clades (Fig. 1). As the AMPK-α amino acid sequences for salmonids are highly conserved, we performed ML phylogenetic analyses at the nucleotide-level, which provides greater phylogenetic signal^19^. Note, this was done for all amino acid trees with any evidence for salmonid-specific paralogues. In the nucleotide level analyses, the salmonid sequences split into two clades representing each of the included species for both AMPK-α1 and AMPK-α2 (Fig. S1). As salmonid sequences branch as the sister clade to northern pike (*Esox lucius*), which did not undergo the ssWGD, we conclude that the salmonid clades are products of ssWGD. We hereafter name these paralogue pairs AMPK-α1A1 and AMPK-α1A2, along with AMPK-α2A1 and AMPK-α2A2. Adding further weight to these inferences, the chromosomal location of the same genes in Atlantic salmon^20^, specifically *AMPK-α1A1* (~53 Mbp along Ssa11) vs. *AMPK-α1A2* (~139Mbp along Ssa01) and *AMPK-α2A1* (~29 Mbp along Ssa10) vs. *AMPK-α2A2* (~22Mbp along Ssa23) matches coordinates of large blocks of duplicate genes that have maintained collinearity since the ssWGD^20^.

### Novel salmonid paralogues of AMPK-β

The AMPK-β subunit tree split into two clades (AMPK-β1 and -β2), represented by all included vertebrate species, as reported elsewhere^6,8^ (Fig. 2). The AMPK-β2 clade followed the same pattern as the AMPK-α clades, with no evidence for retention of teleost-specific paralogues. Again, the salmonid AMPK-β2 sequences are split across different clades representative of different salmonid species (Fig. 2). However, the salmonid AMPK-β2 sequences did not split into two clades (Fig. 2), as to be expected if they originated during the ssWGD event. This was likely an artefact, as the nucleotide level ML analysis, split the salmonid AMPK-β1 sequences into two monophyletic clades, each representing the five included species (Fig. S3). Hence, two AMPK-β2 paralogues were evidently retained from ssWGD, named AMPK-β2A1 and AMPK-β2A2. The chromosomal location of *AMPK-β2A1* (~44 Mbp along Ssa03) and *AMPK-β2A2* (~43 Mbp along Ssa14) are also consistent with an origin from ssWGD^20^.

For the AMPK-β1 clade, there is evidence for two teleost paralogues which are likely the result of the tsWGD. (Fig. 2). Spotted gar is the sister to two teleost clades represented by different lineages, including salmonids (Fig. 2). As a result, the salmonid sequences are named AMPK-β1A and AMPK-β1B, but there is no evidence for salmonid-specific paralogues in either teleost clade (Fig. 2).

### Novel salmonid paralogues of AMPK-γ

The AMPK-γ subunit tree splits into three clades (AMPK-γ1, -γ2 and -γ3), represented by all included vertebrate species, as reported elsewhere^6,8^ (Fig. 3). The AMPK-γ1 clade showed no evidence for teleost-specific paralogues (Fig. 3). Within the AMPK-γ1 clade, the salmonid sequences split into two clades represented by different salmonid species (Fig. 3). The same result was observed in a nucleotide-level ML tree (Fig. S4), indicating that two AMPK-γ1A paralogues were retained from ssWGD, and should be named AMPK-γ1A1 and AMPK-γ1A2. The chromosomal location of *AMPK-γ1A1* (~28Mbp along Ssa22) and *AMPK-γ1A2* (~58Mbp along Ssa12) in Atlantic salmon also supports an origin from the ssWGD^20^.

The AMPK-γ2 clade shows evidence for the retention of teleost-specific paralogues named AMPK-γ2A and AMPK-γ2B (Fig. 3). Further, in each of these teleost clades, the salmonid sequences are split into separate clades characterized by different species (Fig. 3). Our nucleotide level ML trees showed that the salmonid sequences in both teleost AMPK-γ2 clades split into two clades representing the included species (Figs S5, S6). Both the AMPK-2A and −2B salmonid clades branched as sisters to northern pike, suggesting an origin from the ssWGD. The AMPK-γ2A clade sequences were named AMPK-γ2A1 and AMPK-γ2A2, and the AMPK-γ2B clade sequences were named AMPK-γ2B1 and AMPK-γ2B2. The chromosomal location of *AMPK-γ2A1* (~23Mbp along Ssa29) vs. *AMPK-γ2A2* (~70 Mbp along Ssa19), as well as *AMPK-γ2B1* (~26Mbp along Ssa03) vs. *AMPK-γ2B2* (~26Mbp along Ssa14) in Atlantic salmon is again consistent with an origin from the ssWGD^20^.

The AMPK-γ3 subunit clade split into two teleost groups that branched as the sister lineage to spotted gar, suggesting retention of teleost-specific paralogues, named AMPK-γ3A and AMPK-γ3B (Fig. 3). All AMPK-γ sequences from salmonids branched within the AMPK-γ3A clade, indicating loss of AMPK-γ3B in a salmonid ancestor (Fig. 3). There was no evidence for salmonid-specific paralogues of AMPK-γ3A (Fig. 3).

### GH-transgenesis alters constitutive expression of AMPK subunit genes in skeletal muscle

Constitutive differences in transcript level among the three experimental groups were quantified in control (PBS-injected) skeletal muscle for each of the fifteen unique AMPK subunit genes; done using qPCR with primers specific to salmonid-specific paralogues (Table 1). A statistically significant group effect was observed for nine of the genes (Table 1). Several of the subunit genes were significantly upregulated in the GH-transgenic groups when compared to wild-type (WT), with differing effects for transgenic full ration (TF) and transgenic restricted ration (TR) fishes. *AMPK-α2A1* (1.7-fold), *AMPK-β1B* (2.1-fold), *AMPK*-*γ2B1* (4.4-fold) and *AMPK* -*γ2B2* (5.0-fold) were upregulated in TF vs. WT (Table 1). *AMPK-α2A2* (1.9-fold), *AMPK*-*β1A* (1.9-fold), *AMPK-β1B* (1.9-fold) and *AMPK-γ1A1* (1.8-fold) were significantly upregulated in TR vs. WT (Table 1). *AMPK*-*γ2B1* was significantly upregulated in TF vs. TR (4.0-fold), while *AMPK-β1A* (1.5-fold) and *AMPK-γ3A* (1.8-fold) were both downregulated in TF vs. TR (Table 1).

**Table 1:** Constitutive expression changes in AMPK subunit genes between TF, TR and WT coho salmon groups

AMPK subunit gene	*P*-value	TF mean ± s.d.	TR mean ± s.d.	WT mean ± s.d.
*AMPK-α1A1*	0.082	0.56 ± 0.04^A^	0.71 ± 0.13^A^	0.55 ± 0.14^A^
*AMPK-α1A2*	**0.049**	1.03 ± 0.23^A^	1.02 ± 0.17^A^	0.70 ± 0.23^A^
*AMPK-α2A1*	**0.021**	2.38 ± 0.49^A^	1.90 ± 0.36^A,B^	1.38 ± 0.57 ^B^
*AMPK-α2A2^*	**0.031**	7.16 ± 3.79^A,B^	7.53 ± 1.97^A^	4.00 ± 1.14^B^
*AMPK-β1A*	**0.003**	3.81 ± 0.52^B^	5.62 ± 0.96^A^	3.01 ± 1.23^B^
*AMPK-β1B*	**0.001**	0.52 ± 0.07^A^	0.48 ± 0.14^A^	0.25 ± 0.05^B^
*AMPK-β2A1*	0.478	16.59 ± 1.93^A^	20.98 ± 4.52^A^	17.55 ± 8.79^A^
*AMPK-β2A2*	0.284	2.30 ± 0.31^A^	2.61 ± 0.56^A^	2.08 ± 0.61^A^
*AMPK-γ1A1*	**0.012**	0.41 ± 0.06^A,B^	0.54 ± 0.15^A^	0.30 ± 0.08^B^
*AMPK-γ1A2^*	0.325	0.09 ± 0.02^A^	0.17 ± 0.10^A^	0.11 ± 0.03^A^
*AMPK-γ2A1^*	0.071	0.50 ± 0.27^A^	0.41 ± 0.14^A^	0.24 ± 0.09^A^
*AMPK-γ2A2^*	0.070	0.12 ± 0.04^A^	0.33 ± 0.31^A^	0.12 ± 0.05^A^
*AMPK-γ2B1^*	**<0.001**	7.82 ± 2.51^A^	1.96 ± 0.76^B^	1.79 ± 0.82^B^
*AMPK-γ2B2^*	**0.002**	21.84 ± 11.69^A^	8.54 ± 1.38^A,B^	4.38 ± 2.71^B^
AMPK-γ3A*	**0.011**	1.94 ± 0.45^B^	3.57 ± 0.2^A^	3.66 ± 0.5^A^

Transcript levels (mean ± s.d., n = 5) are given for the three experiment groups (PBS-injected, controls) and are quantitatively comparable across groups/genes. For each gene, different superscript letters indicate significant differences across groups. ^‘^’^Highlights genes that required Box-Cox transformation. ^‘*’^Highlights genes where a Kruskal-Wallis test was used.

### Immune status alters AMPK subunit expression in GH-transgenic skeletal muscle

Challenge with two immune stimulants, peptidoglycan (Table 2) and polyinosinic:polycytidylic acid (Poly I:C) (Table 3), intended to generate a pro-inflammatory and antiviral response, respectively, caused marked changes in the expression of several AMPK subunit genes. We observed systematic responses between the GH-transgenic and WT groups, recaptured by significant strain:treatment interaction effects (Tables 2 and 3). In the GH-transgenic groups, immune stimulation almost invariably caused downregulation of AMPK subunit expression, when many of the same genes were upregulated in WT (Tables 2 and 3). Several AMPK subunit genes were affected by immunostimulation in either but not both TF and TR, indicating an effect depending on nutritional status. Interestingly, the same AMPK subunit genes typically showed highly coupled responses to separate peptidoglycan and Poly I:C challenges (compared Tables 2 and 3).

**Table 2:** AMPK subunit gene expression responses of TF, TR and WT coho salmon groups to peptidoglycan stimulation

AMPK subunit gene	*P*-value Treatment	*P*-value Strain:Treatment	TF mean ± s.d.	TR mean ± s.d.	WT mean ± s.d.	TF fold-change	TR fold-change	WT fold-change
*AMPK-α1A1*	0.001	0.033	0.43 ± 0.15^A^	0.42 ± 0.08*^,A^	0.54 ± 0.09^A^		+1.7	
*AMPK-α1A2*	0.943	<0.001	0.55 ± 0.18*^,A^	0.75 ± 0.19*^,A,B^	1.44 ± 0.3*^,B^	+1.9	+1.4	−2.0
*AMPK-α2A1*	0.238	0.020	1.50 ± 0.34^A^	1.76 ± 0.57^A^	1.78 ± 0.45^A^			
*AMPK-α2A2^*	0.005	<0.001	3.12 ± 1.09*^,A^	3.82 ± 0.73*^,A,B^	5.81 ± 0.88*^,B^	+2.3	+2.0	−1.4
*AMPK-β1A*	0.494	0.016	3.19 ± 0.98^A^	4.37 ± 0.61^A^	4.19 ± 0.89^A^			
*AMPK-β1B*	0.792	<0.001	0.26 ± 0.17*^,A^	0.38 ± 0.10^A,B^	0.58 ± 0.10*^,B^	+2.0		−2.3
*AMPK-β2A1*	0.516	0.416	18.47 ± 1.82^A^	17.34 ± 3.68^A^	15.99 ± 3.09^A^			
*AMPK-β2A2*	0.000	0.315	1.23 ± 0.34*^,A^	1.31 ± 0.31*^,A^	1.37 ± 0.39^A^	+1.9	+2.0	
*AMPK-γ1A1*	0.340	0.001	0.28 ± 0.10^A^	0.35 ± 0.06^A^	0.51 ± 0.15^A^			
*AMPK-γ1A2*	0.207	0.155	0.15 ± 0.09^A^	0.13 ± 0.03^A^	0.18 ± 0.05^A^			
*AMPK-γ2A1^*	0.037	0.006	0.18 ± 0.06*^,A^	0.31 ± 0.08^A^	0.30 ± 0.08^A^	+2.7		
*AMPK-γ2A2^*	0.065	0.197	0.14 ± 0.13^A^	0.10 ± 0.06^A^	0.11 ± 0.06^A^			
*AMPK-γ2B1^*	0.136	<0.001	1.07 ± 0.29*^,A^	2.31 ± 0.60^A^	5.34 ± 1.82^B^	+7.3		−3.0
*AMPK-γ2B2^*	0.239	<0.001	5.89 ± 1.25*^,A^	8.70 ± 4.97^A^	24.77 ± 3.57*^,B^	+3.7		−5.7
*AMPK-γ3A^*	0.609	<0.001	3.26 ± 0.48^A^	3.71 ± 0.86^A^	2.38 ± 0.89^A^			

Transcript levels (mean ± s.d., n = 5) are given for the three experiment groups (peptidoglycan-injected) and are quantitatively comparable across groups/genes. ^‘*’^Indicates a significant change in expression due to treatment; for these genes, fold change values are shown, which were calculated by dividing the mean peptidoglycan treatment by the mean control transcript levels (Table 1); + and − symbols depict up and downregulation, respectively. For each gene, different superscript letters indicate significant differences across groups for the peptidoglycan treatment samples. ^‘^’^Highlights genes that required a Box-Cox transformation.

**Table 3:** AMPK subunit gene expression responses of TF, TR and WT coho salmon groups to Poly I:C stimulation

AMPK subunit gene	*P*-value Treatment	*P*-value Strain:Treatment	TF mean ± s.d.	TR mean ± s.d.	WT mean ± s.d.	TF fold-change	TR fold-change	WT fold-change
*AMPK-α1A1*	0.011	<0.001	0.35 ± 0.11^A^	0.42 ± 0.07*^,A^	0.67 ± 0.12^B^		+1.7	
*AMPK-α1A2*	0.196	<0.001	0.49 ± 0.17*^,A^	0.57 ± 0.22*^,A^	1.16 ± 0.28*^,B^	+2.1	+1.8	−1.7
*AMPK-α2A1*	0.162	0.036	1.66 ± 0.64^A^	1.60 ± 0.42^A^	1.82 ± 0.31^A^			
*AMPK-α2A2^*	0.382	<0.001	3.04 ± 0.64*^,A^	3.19 ± 1.02*^,A^	6.75 ± 2.58^B^	+2.4	+2.4	
*AMPK-β1A*	0.218	0.003	4.01 ± 1.07^A^	3.91 ± 0.59^A^	5.19 ± 1.81*^,A^			−1.7
*AMPK-β1B*	0.482	0.006	0.35 ± 0.21^A^	0.42 ± 0.16^A^	0.51 ± 0.15^A^			
*AMPK-β2A1*	0.936	0.279	18.34 ± 3.59^A^	15.62 ± 4.19^A^	18.7 ± 6.42^A^			
*AMPK-β2A2*	0.336	0.016	1.33 ± 0.33*^,A^	1.49 ± 0.14*^,A^	2.15 ± 0.62^A^	+1.7	+1.8	
*AMPK-γ1A1*	0.311	0.001	0.29 ± 0.12*^,A^	0.30 ± 0.09^A,B^	0.55 ± 0.20*^,B^	+1.4		−1.8
*AMPK-γ1A2^*	0.115	0.157	0.09 ± 0.07^A^	0.07 ± 0.04^A^	0.18 ± 0.13^A^			
*AMPK-γ2A1*	0.528	0.071	0.31 ± 0.15^A^	0.28 ± 0.14^A^	0.40 ± 0.11^A^			
*AMPK-γ2A2*	0.266	0.032	0.09 ± 0.08^A^	0.13 ± 0.11^A^	0.25 ± 0.19^A^			
*AMPK-γ2B1^*	0.003	<0.001	1.34 ± 0.53*^,A^	1.27 ± 0.33^A^	3.34 ± 1.40^B^	+5.9		
*AMPK-γ2B2^*	0.065	<0.001	6.42 ± 2.09*^,A^	5.76 ± 2.39^A^	15.5 ± 4.28*^,B^	+3.4		−3.5
*AMPK-γ3A*	0.481	0.001	3.51 ± 0.81*^,A^	2.59 ± 0.42^A^	2.32 ± 0.74^A^	−1.8		

Transcript levels (mean ± s.d., n = 5) are given for the three experiment groups (Poly I:C-injected) and are quantitatively comparable across groups/genes. ^‘*’^Indicates a significant change in expression due to treatment; for these genes, fold change values are shown, which were calculated by dividing the mean Poly I:C treatment by the mean control transcript levels (Table 1); + and − symbols depict up and downregulation, respectively. For each gene, different superscript letters indicate significant differences across groups for the Poly I:C treatment samples. ^‘^’^Highlights genes that required a Box-Cox transformation.

Seven AMPK subunit genes decreased in TF in response to peptidoglycan, namely *AMPK-α1A2*, *AMPK*-*α2A2*, *AMPK-β1B*, *AMPK-β2a2*, *AMPK*-*γ2A1*, *AMPK*-*γ2B1* and *AMPK*-*γ2B2* (Table 2). TR shared the same response for *AMPK-α1A2*, *AMPK*-*α2A2* and *AMPK-β2A2*, while also showing a distinct downregulation of *AMPK-α1A1* (Table 2). In WT, five of the same genes showed upregulation to peptidoglycan, namely *AMPK-α1A2*, *AMPK*-*α2A2*, *AMPK*-*β1B*, *AMPK*-*γ2B1* and *AMPK-γ2B2* (Table 2). Additionally, several genes had significantly lower expression comparing the GH-transgenic groups with WT in the peptidoglycan treatment samples, namely AMPK*-α1A2* (TF only), *AMPK*-*α2A2* (TF only), *AMPK-β1B* (TF only), *AMPK*-*γ2B1* (TF and TR) and *AMPK*-*γ2B2* (TF and TR) (Table 1).

Six AMPK subunit genes decreased in TF in response to Poly I:C challenge, namely *AMPK-α1A2*, *AMPK*-*α2A2*, *AMPK*-*β2A2*, *AMPK* -*γ2B1* and *AMPK*-*γ2B2* (Table 3). TR shared the same downregulation response for *AMPK-α1A2, AMPK-α2A2* and *AMPK-β2A2* while also exhibiting a separate downregulation for *AMPK-α1A1* (Table 3). *AMPK-γ3A* was the single gene that showed upregulation in either GH-transgenic group (TF) following Poly I:C treatment (Table 3). For WT, four AMPK subunit genes were upregulated by Poly I:C: *AMPK-α1A2*, *AMPK-β1A*, *AMPK-γ1A1* and *AMPK-γ2B2* (Table 3). Several genes had significantly lower expression comparing the GH-transgenic groups to WT after Poly I:C treatment: *AMPK-α1A1* (TF and TR)*, AMPK-α1A2* (TF and TR)*, AMPK-α2A2* (TF and TR)*, AMPK-γ1A1* (TF only), *AMPK-γ2B1* (TF and TR) and *AMPK* -*γ2B2* (TF and TR) (Table 3).

## Discussion

The first objective of this study was achieved by establishing the complete set of AMPK subunit genes retained in the salmonid lineage from the tsWGD and ssWGD events. Phylogenetic analyses revealed genetic expansions of all three AMPK subunits as a product of the tsWGD event, confirming past findings^6,8^. Furthermore, we identified additional salmonid-specific paralogues due to the ssWGD, an event that has been associated with an high overall paralogue retention rate^20,21^, which nonetheless shows variability across different gene families (e.g.^38^). Evidently, salmonids retain at least fifteen transcriptionally active and presumably functional genes encoding AMPK complex proteins, four for AMPK-α, four for AMPK-β and seven for AMPK-γ. This represents a significant expansion compared to humans (seven genes) and other teleosts such as zebrafish (ten genes). Our phylogenetic analyses will be useful for researchers wishing to further explore the salmonid AMPK system, providing a reference to robustly interpret signals of functional divergence and conservation among paralogues.

The second objective of the study was achieved by determining the extent to which AMPK genes are transcriptionally regulated by growth and immune status in coho salmon skeletal muscle, testing the hypothesis that AMPK plays a role in the regulation of resource allocation across different physiological systems. We confirmed that AMPK is extensively transcriptionally regulated by GH-transgenesis, with some variation between TF and TR, and by immune stimulation from both bacterial and viral mimics, with striking differences between GH-transgenics and WT. This is consistent with our past work on the same coho salmon muscle samples, where GH-transgenesis was shown to alter the expression responses of both the innate immune system, as well as the GH and IGF pathways, in comparison to WT^27^. However, it is important to emphasize that our study provides no information on changes in AMPK regulation at the level of total protein abundance, or phosphorylation status, both which may fail to correlate with transcript levels.

Changes in baseline mRNA expression for AMPK subunit genes usually involved upregulation in TF and/or TR compared to WT, including for *AMPK-α2A1* (TF only)*, AMPK-γ2B1A* (TR only) and *AMPK-γ2B2* (TF only). The AMPK-α subunit contains the main phosphorylation site leading to AMPK activity making it a strong candidate for mediating the effects of GH-transgenesis. Indeed, enhancements in *AMPK-γ2* expression lead to a concomitant AMPK activity escalation in mice^39^. Our results are thus contrary to the assumption that increased AMPK activity would lead to the scaling down of anabolic pathways and increases in catabolic pathways, considering the increased growth phenotype of TF. However, in mouse, overexpression of *AMPK-α* and *γ* subunits can lead to the accumulation of glycogen^40,41^, which may support faster growth rate. Perhaps related to this observation, our previous proteomic study highlighted pervasive changes in skeletal muscle carbohydrate metabolism in the same GH-transgenic animals when compared to WT, with GH-transgenics showing an increased abundance of proteins involved in glycolysis, glycogenolysis and gluconeogenesis^42^. Differing constitutive expression of both *AMPK-γ2B* paralogues between TF and TR may be caused by differences in growth rate, as induced by elevated endocrine IGF-I in TF salmon^30^ compared to both the TR and WT groups. The additional increase in expression of *AMPK-β1A* in TR compared to WT, suggests a direct modification due to GH, independent of IGF-I mediated effects, given that IGF-I levels are comparable in TR and WT^30^. Contrary to these increases, *AMPK-γ3A* was significantly lower in TF compared to TR. In mammals, AMPK-γ3 is predominantly expressed in skeletal muscle^43^, where it is important for glycogen metabolism, indicating that decreased expression of *AMPK-γ3* leads to glycogen accumulation^44–46^, with potential implications for other glycogen driven anabolic pathways. These findings highlight disruption of WT AMPK signalling due to GH-transgenesis across all three AMPK subunit families.

The most established function of AMPK is sensing cellular energy status, leading to modifications of metabolic pathways, but AMPK also has known roles in regulating immune responses^47–50^. The transcriptional response of innate immune markers (previously measured for the same set of samples used in this study) highlighted a strong impact of GH-transgenesis on immune function in coho salmon skeletal muscle^27^. After exposure to Poly I:C, no appreciable antiviral response was present in TF, whereas robust upregulation of gene markers for type-I interferon signalling occurred in WT fish^27^. Treatment with peptidoglycan led to an attenuated upregulation of inflammatory cytokines in TF fish compared to WT fish^27^. Interestingly, in TR the same immune marker genes showed an intermediate response to immune stimulation, suggesting that accelerated growth rate was the primary cause of attenuated muscle immune function in TF^27^. Moreover, both TF and TR showed complex alterations in the expression of GH and IGF pathway genes relative to WT in response to immune stimulation^27^. Such alterations in the relationship between growth and immune systems in skeletal muscle suggest an accompanying requirement for a shift in AMPK function as the main cellular energy sensor. Our data provides support for this concept through complex differences in the expression of AMPK subunit genes as a function of growth and immune status.

A common set of AMPK-α subunit genes experienced downregulation of mRNA expression in both TF and TR vs. WT in response to peptidoglycan and Poly I:C. Interestingly, many of the same genes were upregulated in WT, which may serve to activate catabolic AMPK pathways needed for energetic reallocation towards a robust immune response^48,51^. Whatever the underlying cause for upregulation in WT fish, the reciprocal downregulation observed in the GH-transgenic strain suggests a disruption of the normal AMPK system transcriptional response to immunological challenge. The AMPK-α subunit contains the main site for phosphorylation at Thr172, leading to increases in AMPK activity by more than 100-fold^52^. The observed reduced expression of AMPK-α subunit genes during immune stimulation could lead to disruption of signalling pathways in the cytosol, transcription and gene expression in the nucleus and a reduced ability to uptake glucose into skeletal muscle^46,53^. Additionally, AMPK-α1 has been shown to be crucial for AMPK activation due to glucose deprivation, including from cytotoxic T lymphocytes^51^. Alterations to such functions could potentially impact the ability of the AMPK complex to sense cellular energy status with impacts on the allocation of energetic investment into growth and immune functions. Both immune stimulants caused reduced expression of *AMPK-β2A2* in TF and TR, while *AMPK-β1B* also showed significantly lower expression for TF compared to WT in response to peptidoglycan. This decline in AMPK-β subunit gene expression may lead to overall reduction in AMPK activity due to the importance of the AMPK-β subunit in AMP binding and subsequent activation of AMPK via phosphorylation^54^. Glycogen serves an important role inhibiting AMPK activation through binding of the β subunit^55^, indicating that the observed decrease in AMPK-β expression in TF could lead to an impaired ability for AMPK to respond properly to excess glycogen, thus potentially reducing the ability of the skeletal muscle tissue to produce a coordinated shift into a required catabolic state for a proper immune response. The trend of decreased AMPK subunit expression in GH-transgenic salmon continued with AMPK-γ, highlighted by reductions of *AMPK-γ2B1* and *−2B2* in response to both peptidoglycan and Poly I:C for TF compared to WT. Similar to AMPK-α, decreased expression of *AMPK-γ2B1* and *−2B2* in TF exposed to peptidoglycan and Poly I:C is compounded by the heightened expression of the WT group in response to either immune stimuli. However, the importance of AMPK-γ to enzyme activity cannot be understated, as it is the primary binding site for AMP and ATP used by the AMPK complex to determine cellular energy status^56,57^. A reduced ability to sense dynamic cellular energetic status could contribute to an attenuation of cross-talk between the growth and immune systems linked to excess GH, causing an attenuated immune response in GH-transgenic fish.

Given the increased number of AMPK subunits present in the salmonid genome, it is important to contrast expression responses among salmonid-specific paralogues. For example, paralogue pairs for both AMPK-α1A and AMPK-γ2B showed similar expression changes in response to peptidoglycan and Poly I:C in all three strains, suggesting maintenance of functional elements controlling their transcriptional regulation. However, for AMPK-α2A and AMPK-β2A, single genes in each paralogue pair (*AMPK-α2A2* and *AMPK-β2A2*, respectively) were significantly altered by the same immune challenges, suggesting loss of immune-responsive elements in one gene duplicate, perhaps by sub-functionalization. In addition, in several cases the constitutive mRNA expression levels of AMPK subunit genes was notably different among paralogue pairs, including for AMPK-β1A, AMPK-β2A, AMPK-γ1A and AMPK-γ2B. The overall importance of divergent or conserved expression profiles between many salmonid-specific paralogues remains unclear. However, as no two salmonid-specific AMPK subunit paralogues act in a completely antagonistic fashion, the most likely scenario is that paralogues are acting in a cooperative and/or synergistic fashion. These findings further emphasise an ongoing need to properly define the functional physiological importance of paralogue expression divergence following ssWGD, which represents an important ongoing study area^18^.

In conclusion, salmonid fishes retain an expanded set of genes encoding the AMPK-α, β and γ subunits, owing to a history of whole genome duplication events. Using a coho salmon model that allowed us to contrast the impact of immune stimulation on GH-transgenic vs. wild-type fish, we demonstrate that genes from each AMPK subunit family, which together are required for full AMPK activity^58^, show coordinated transcriptional responses to changes in growth and immune status in skeletal muscle. While additional work is needed to understand how such complex mRNA responses correspond to AMPK function via changes in the translation (and post-translational status) of individual AMPK subunit paralogues, our findings support a role for the AMPK system in balancing investment between growth and immunity in fish.

## Materials and Methods

### Phylogenetic analyses of AMPK subunits

Protein-level phylogenetic analyses were performed separately for the AMPK-α, -β and -γ subunits, which represent different gene families with unique evolutionary histories (e.g.^6,8^). As a start point, the complete set of AMPK-α, -β and -γ family members from human (*Homo sapiens*) (AMPK-α1: NP_006242.5; AMPK-α2: NP_006243.2; AMPK-β1: NP_006244.2; AMPK-β2: NP_005390.1; AMPK-γ1: NP_002724.1; AMPK-γ2: NP_057287.2; AMPK-γ3: NP_059127.2) were used as queries in BLASTp^59^ searches against the NCBI non-redundant protein database to extract putative orthologues from a range of vertebrates, including salmonids with available reference genome sequences. The collected protein sequences for AMPK-α, (*n* = 38), AMPK-β (*n* = 42) and AMPK-γ (*n* = 65) were separately aligned using MAFFT v7^60^ with default settings followed by quality filtering (default settings) using the GUIDANCE2 algorithm^61^ through the GUIDANCE server^62^. This led to the following number of aligned amino acid positions: 506, 231 and 327 for the AMPK-α, -β and –γ datasets, respectively. The IQ-TREE maximum likelihood (ML) approach^63^ and server^64^ were used to determine the best-fitting amino acid substitution model (JTT + G4 in each case) separately for the three individual subunit alignments and build consensus ML trees with the same model, using 1,000 ultrafast bootstrap replicates^65^.

In addition, we performed six separate nucleotide-level phylogenetic analyses, one per each putative pair of salmonid-specific paralogues identified in the amino acid ML trees. In these analyses, all salmonid-specific paralogues from each included salmonid species were aligned along with the relevant northern pike (*Esox lucius*) orthologue (closest outgroup species to the ssWGD event)^25^ using MAFFT v7, as described above. Subsequently, ML trees were built in IQ-TREE as described above except incorporating the best-fitting model of nucleotide substitution and without alignment filtering. Consensus ML trees were visualized and edited using FigTree v1.4.3 (http://tree.bio.ed.ac.uk/software/figtree/).

### Primer design and quantitative PCR

Details of all primer pairs used for quantitative PCR (qPCR) are provided in Table S1. New primers matching to fifteen unique AMPK subunit genes identified in salmonids (see Results section) were designed for coho salmon (Table S1). We designed primers in regions that were as conserved as possible across salmonid species and distinguishing among any identified pairs of salmonid-specific paralogues (noted in Table S1). Design of new primers was aided by the use of NetPrimer (PREMIER Biosoft), which predicted no self- or cross-dimers. Primers were also predicted to either span an exon-exon boundary or be positioned in different exons, based on cross-referencing with the reference coho salmon genome (NCBI accession; GCA_002021735.1).

The cDNA samples used as the template for all qPCR analysis reported in this study were prepared during a past study^27^ (full methods described therein). Briefly, three coho salmon groups were used at matched body sizes, which required sampling at different ages due to differences in growth rate: i) satiation-fed wild-type (WT) fish aged 19 months; ii) satiation-fed GH-transgenic fish aged 6 months (transgenic full ration: ‘TF’); iii) GH transgenic fish aged 17 months fed a restricted wild-type ration (transgenic restricted ration: ‘TR’). Fish were sampled either 30 h after Poly I:C injection to mimic a viral infection (*n = 5* cDNA biological replicates per TF, TR and WT), 30 h after peptidoglycan injection to mimic a bacterial infection (*n = 5* cDNA samples per TF, TR and WT) or 30 h after PBS injection as a control (*n = 5* cDNA samples per TF, TR and WT).

An Mx3005P qPCR System (Agilent Technologies) was used to measure transcript levels of all target genes. Each reaction contained 5 µl of 1:100 cDNA (corresponding to 2.5 ng of reverse-transcribed total RNA), 500 nM sense/antisense primers and 7.5 µl Brilliant III Ultra-Fast SYBR Green (Agilent Technologies), totalling 15 µl per reaction. Conditions for thermal cycling were 1x cycle of 95 °C for 3 min, followed by 40x cycles of 95 °C for 20 seconds and 64°C for 20 seconds. The presence of a single product for all assays was confirmed with a dissociation analysis (thermal gradient from 55°C to 95°C). Technical duplicates were performed for each assay, and each 96-well plate contained all samples for each target gene. Duplicate no-template controls (cDNA replaced with water) were used in each plate. LinRegPCR was used to determine the efficiency of each qPCR assay following published recommendations^66^. Two reference genes (*RpL13* and *ACTB*) selected for data normalization were shown to be the most suitable among a panel of five candidates in a previous study using the same samples (primer sequences are provided therein)^27^. The program GenEx (MultiD Analyses AB) was used for efficiency correction and normalization to arbitrary transcript levels on a quantitatively comparable scale for all AMPK subunit genes measured.

### Statistical analyses

R-studio v1.0.136 (Rstudio, Boston, MA) interfacing with R v3.3.2 (“Sincere Pumpkin Patch”) was used for statistics and graphical functions, using the normalized qPCR transcript values. Two genes had non-detectable *Ct* values (*AMPK-γ2A1* and *AMPK-γ2A2* for 2 and 7 out of 45 samples, respectively); these missing values were imputed^67^ to increase statistical power, using missForest^68^. A linear model was fit to each AMPK subunit gene to determine the overall effect of strain (WT vs. TF vs. TR: fixed factors), treatment (PBS vs. Poly I:C or PBS vs. peptidoglycan: fixed factors) and, where relevant, the strain:treatment interaction. Where statistically significant overall effects were observed (*P* < 0.05), pair-wise comparisons using Tukey’s method were completed to determine differences among the different levels in the model. The model residuals were tested for normality (Anderson-Darling test) and homogeneity in variance (Levene’s test) as well as through visual assessment. When the data failed to meet these assumptions, Box-Cox transformations were completed using the ‘car’ package^69^, which recovered normality and homogeneity in variance for all comparisons, except the strain comparison for *AMPK-γ3A*, which required a Kruskal-Wallis test.

## Supplementary information

Supplementary Information

Supplementary Datasets

## Data Availability

Sequence alignments, including accession numbers for all individual sequences, are provided as supplementary datasets-1 (AMPK-α), −2 (AMPK-β) and −3 (AMPK-γ). Nucleotide alignments for the salmonid-specific paralogues, including accession numbers for all individual sequences, are provided as supplementary datasets-4 (AMPK-α1), −5 (AMPK-α2), −6 (AMPK-β2), −7(AMPK-γ1), −8(AMPK-γ2A) and −9 (AMPK-γ2B). Additionally, mean transcript levels and full output from the statistical models are available as supplemental tables (Tables S2 & S3).
