# Supplementary material for: The AMPK system of salmonid fishes was expanded through genome duplication and is regulated by growth and immune status in muscle
Source: Sci Rep. 2019 Jul 8;9:9819. doi: 10.1038/s41598-019-46129-4 (PMC6614447; doi:10.1038/s41598-019-46129-4)
Supplement: Supplementary file 1 — Supplementary Information [file 41598_2019_46129_MOESM1_ESM.docx]

*Supplementary Information*:

**The AMPK system of salmonid fishes was expanded through genome duplication and is regulated by growth and immune status in muscle**

Dwight R. Causey, Jin-Hyoung Kim, Robert H. Devlin, Samuel A.M. Martin & Daniel J. Macqueen

Includes: Fig. S1 to S3 and Table S1

**Supplementary Figures:**


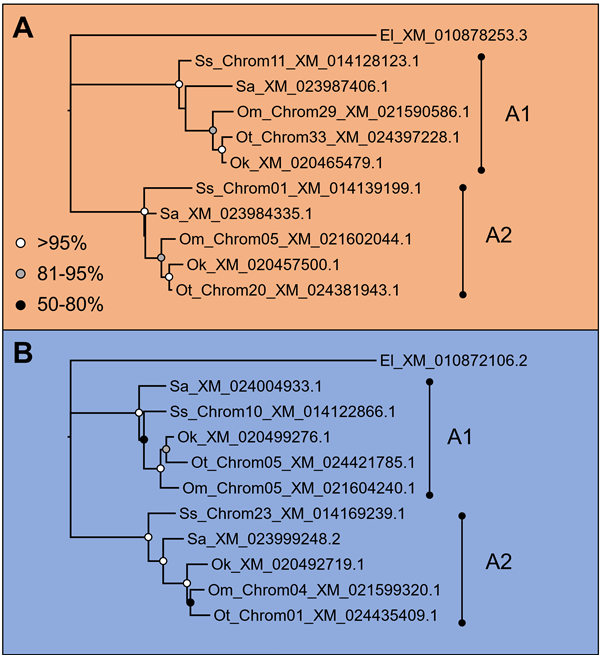


**Fig. S1**. Nucleotide-level maximum likelihood phylogenetic analysis of AMPK-α subunits (**A**: AMPK-α1, **B:** AMPK-α2) which showed evidence of salmonid-specific paralogues in the main amino acid-level analysis (Fig. 1, main text). Species abbreviations: northern pike *Esox lucius* (“El”, a sister lineage to salmonids that did not undergo ssWGD, Arctic charr *Salvelinus alpinus* (“Sa”), Atlantic *salmon Salmo salar* (“Ss”), rainbow trout *Oncorhynchus mykiss* (“Om”), Chinook salmon *Oncorhynchus tshawytscha* (“Ot”), and coho salmon *Oncorhynchus kisutch* (“Ok”). Bootstrap branch support values are shown as circles on each node. Chromosomal locations for salmonid genes are provided when available. Accessions numbers are provided for all sequences.


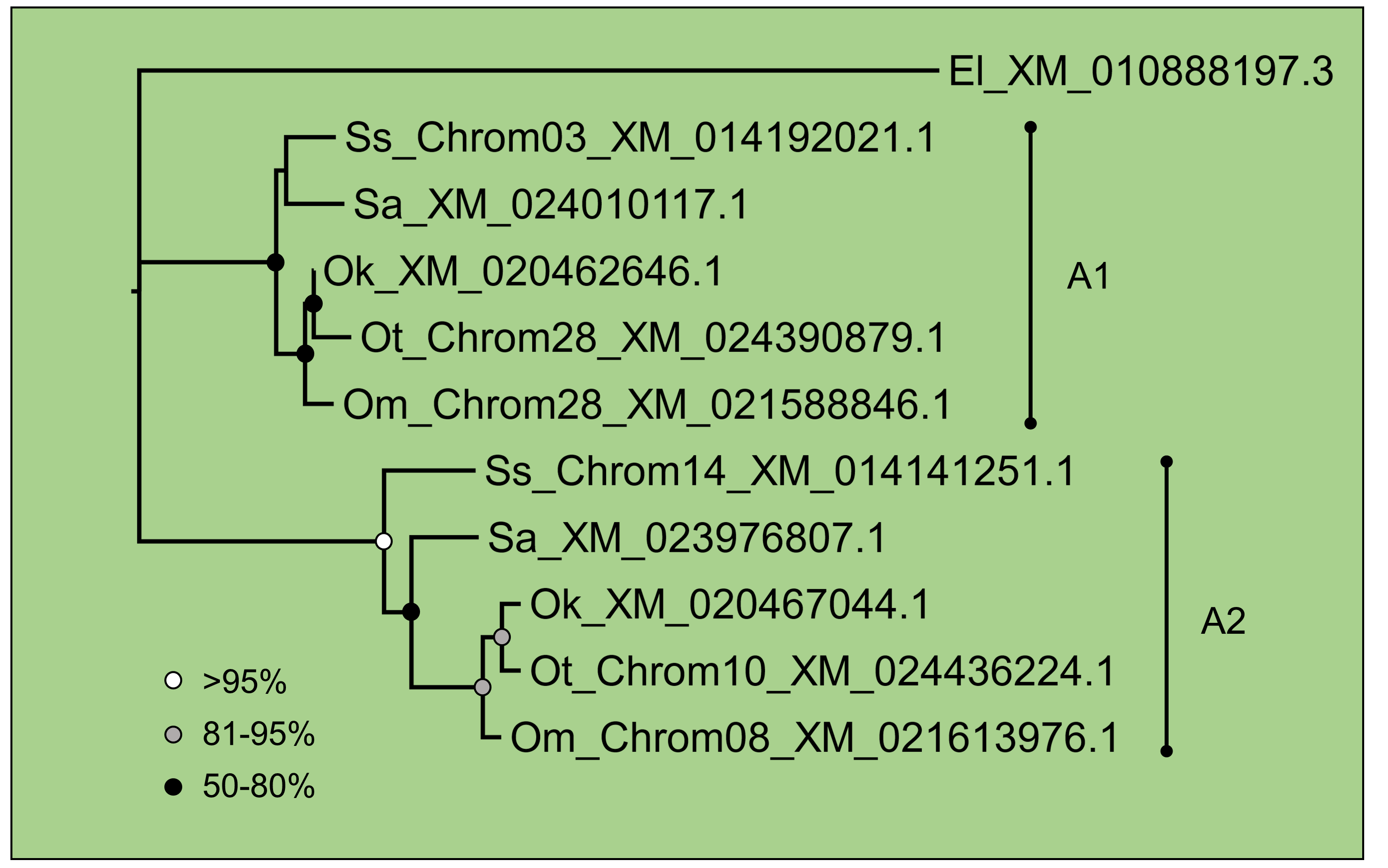


**Fig. S2**. Nucleotide-level maximum likelihood phylogenetic analysis of any AMPK-β subunits (AMPK-β2) which showed evidence of salmonid-specific paralogues in the main amino acid-level analysis (Fig. 2, main text). Species abbreviations: northern pike *Esox lucius* (“El”, a sister lineage to salmonids that did not undergo ssWGD, Arctic charr *Salvelinus alpinus* (“Sa”), Atlantic *salmon Salmo salar* (“Ss”), rainbow trout *Oncorhynchus mykiss* (“Om”), Chinook salmon *Oncorhynchus tshawytscha* (“Ot”), and coho salmon *Oncorhynchus kisutch* (“Ok”). Bootstrap branch support values are shown as circles on each node. Chromosomal locations for salmonid genes are provided when available. Accessions numbers are provided for all sequences.

**
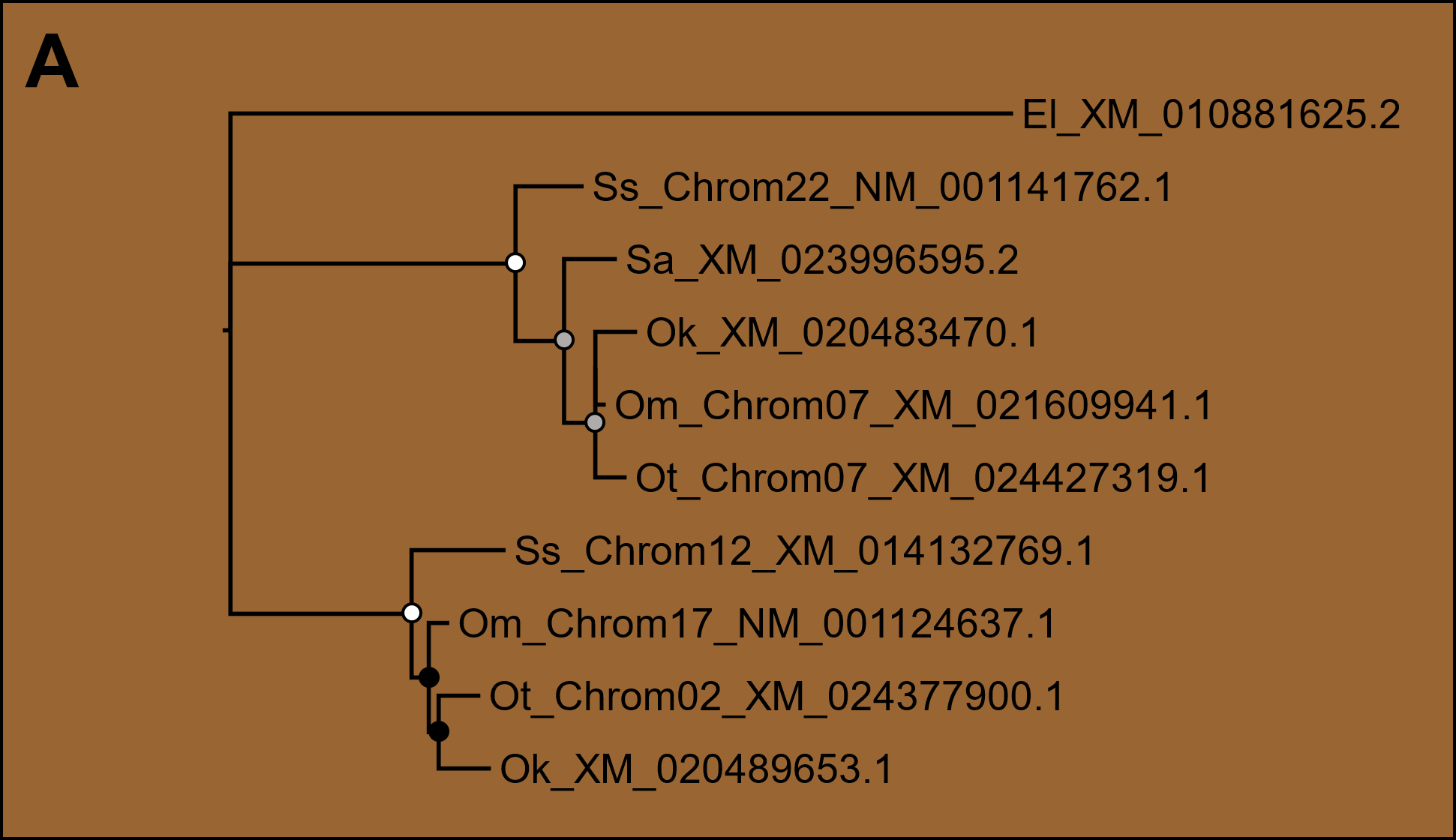
**

**
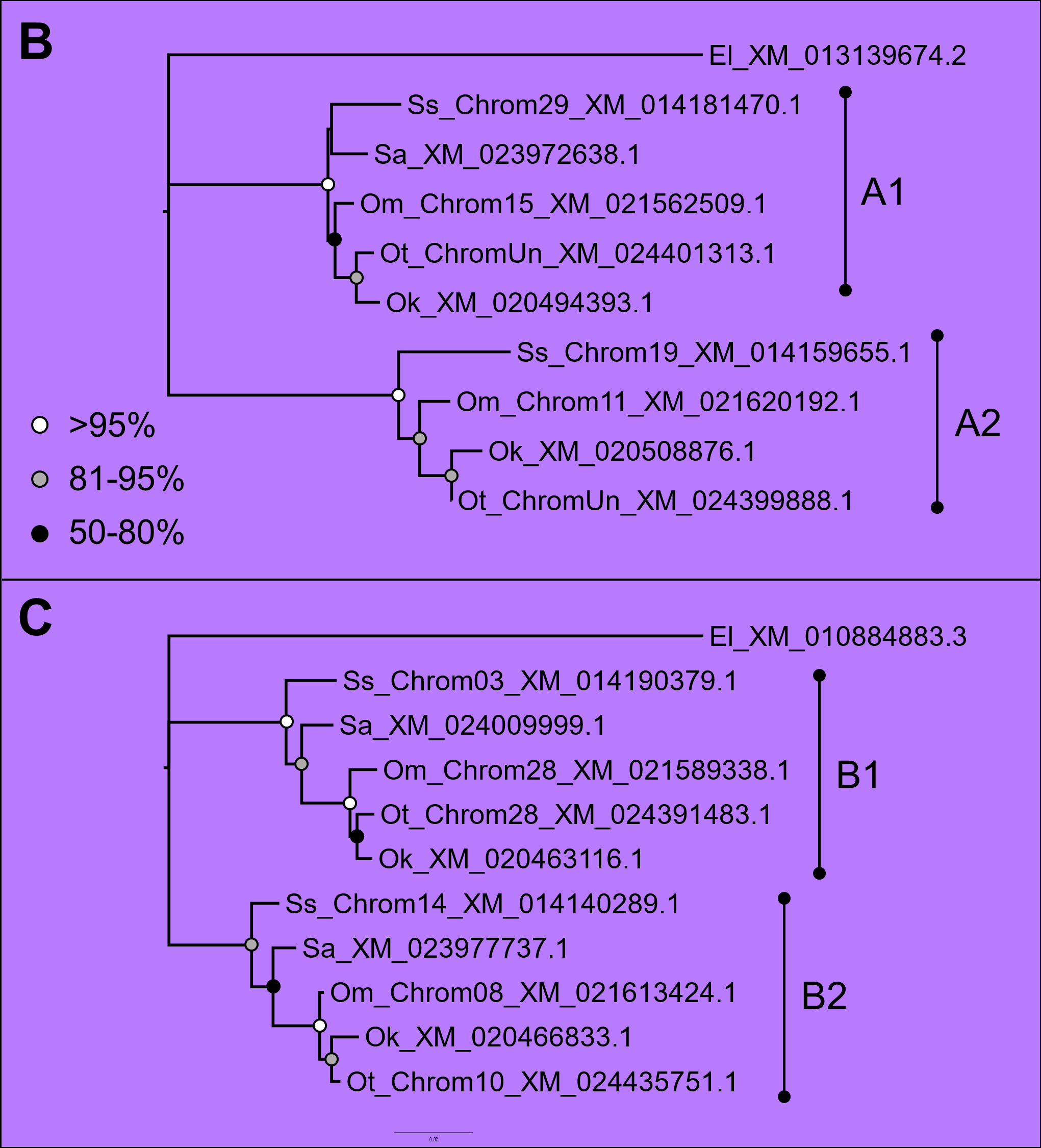
**

**Fig. S3**. Nucleotide-level maximum likelihood phylogenetic analysis of any AMPK-γ subunits (**A**: AMPK- γ1, **B**: AMPK- γ2A, **C**: AMPK- γ2B) which showed evidence of salmonid-specific paralogues in the main amino acid-level analysis (Fig. 2, main text). Species abbreviations: northern pike *Esox lucius* (“El”, a sister lineage to salmonids that did not undergo ssWGD, Arctic charr *Salvelinus alpinus* (“Sa”), Atlantic *salmon Salmo salar* (“Ss”), rainbow trout *Oncorhynchus mykiss* (“Om”), Chinook salmon *Oncorhynchus tshawytscha* (“Ot”), and coho salmon *Oncorhynchus kisutch* (“Ok”). Bootstrap branch support values are shown as circles on each node. Chromosomal locations for salmonid genes are provided when available. Accessions numbers are provided for all sequences.

**Table S1**. Primers used in the study for quantitative PCR analysis of AMPK subunit encoding genes

| **Gene** | **Accession** | **Sense Primer** | **Tm**  **(^o^C)** | **Anti-sense primer** | **Tm (^o^C)** | **Efficiency (%)** | **Product size (bp)** |
| --- | --- | --- | --- | --- | --- | --- | --- |
| *AMPK-α1A1* | XM_020465479 | CTGGTGCTTCGACTTCAGGG | 68.6 | GC------------GGCGGTCAGGTGCTGGT | 60.4 | 90.5% | 142 |
| *AMPK-α1A2* | XM_020457500 | TTGTTCCCAGAGGACCCGTC | 61.8 | GTGCTGATCGTCCAAGAAGG | 57.7 | 91.6% | 236 |
| *AMPK-α2A1* | XM_020499276 | CGACCCATTGAAGAGAGCC | 57.6 | CCAGAACTGTGGAGTCATAAGACA | 58.7 | 91.7% | 113 |
| *AMPK-α2A2* | XM_020492719 | CCCGCTCAAGAGGGCTAC | 57.9 | CAGAATGGTGGAGTCATAGGAAG | 58.2 | 92.1% | 110 |
| *AMPK-β1A* | XM_020470682 | GCAGGTCAGGGTGAGAGGC | 59.9 | CGGAACACAGTGGGCTTGG | 61.8 | 89.5% | 225 |
| *AMPK-β1B* | XM_020458008 | GAGAGGACATCAAGGCTCCAC | 58.4 | TAGCTGGTTGGTTACAACAGGC | 59.7 | 95.3% | 295 |
| *AMPK-β2A1* | XM_020462646 | GACAGCAGAGGCAGCGACA | 60.1 | CCCAGTTGTTGAAGGAGCCC | 61.7 | 89.6% | 225 |
| *AMPK-β2A2* | XM_020467044 | CGCTCTGACAGCAGA------GACAAAG | 60.3 | TCACCAGGTCATCCAGGTCG | 61.1 | 93.6% | 145 |
| *AMPK-γ1A1* | XM_020483470 | TAACGCAAGTCTGTATGATGCTGTA | 60.0 | GATTCCCAGTGCTGTGTAGAGG | 58.8 | 92.8% | 255 |
| *AMPK-γ1A2* | XM_020489653 | CAATGAAAGTCTGTATGACGCC | 57.5 | AATGTTCCAATGCCCAGTTCC | 61.2 | 92.0% | 200 |
| *AMPK-γ2A1* | XM_020494393 | GGAGGAACATCAGATTGAAACA | 56.9 | GTGGTAGGTCCCGATGGTTAGT | 59.7 | 88.4% | 279 |
| *AMPK-γ2A2* | XM_020508876 | AATATATGAGCTGGAGGAGCATAAA | 59.2 | GGTAGGTGCCGATGGTCAGC | 62.5 | 93.4% | 290 |
| *AMPK-γ2B1* | XM_020463116 | CGCTTTATGAAGTCCCATAGC | 57.2 | ACAAAACACTGCCTCTCCGAG | 59.4 | 92.1% | 156 |
| *AMPK-γ2B2* | XM_020466833 | CGCTTTATGAAGTCCCACTGC | 60.0 | CACTGCCTCTCTGAATCCCAT | 58.9 | 89.4% | 152 |
| *AMPK-γ3A* | XM_020504481 | GCACAAGCGAATCCTCAAGTTT | 61.2 | ACACAGACAGGGCATCGTAGAC | 59.0 | 91.2% | 166 |

Red font indicates base differences between putative ssWGD paralogues; “-” indicates a deletion distinguishing ssWGD paralogues; primer melting temperature (Tm) was calculated using NetPrimer (PREMIER Biosoft); The efficiency of each assay was calculated using LinRegPCR (see Methods)
